# Self-limiting COVID‐19-associated Kikuchi‐Fujimoto disease with heart involvement: case-based review

**DOI:** 10.1007/s00296-021-05088-8

**Published:** 2022-01-13

**Authors:** Anna Masiak, Amanda Lass, Jacek Kowalski, Adam Hajduk, Zbigniew Zdrojewski

**Affiliations:** 1 Department of Internal Medicine, Connective Tissue Diseases and Geriatrics, Medical University of Gdansk, ul. Dębinki 7, 80-952 Gdańsk, Poland; 2 Department of Pathomorphology, Medical University of Gdansk, Gdańsk, Poland

**Keywords:** Kikuchi-Fujimoto disease, COVID-19, Lymphadenopathy, Myocarditis

## Abstract

**Background:**

The association between COVID-19 infection and the development of autoimmune diseases is currently unknown, but there are already reports presenting induction of different autoantibodies by SARS-CoV-2 infection. Kikuchi-Fuimoto disease (KFD) as a form of histiocytic necrotizing lymphadenitis of unknown origin.

**Objective:**

Here we present a rare case of KFD with heart involvement after COVID-19 infection. To our best knowledge only a few cases of COVID-19-associated KFD were published so far. Based on presented case, we summarize the clinical course of KFD and its association with autoimmune diseases, as well we discuss the potential causes of perimyocarditis in this case.

**Methods:**

We reviewed the literature regarding cases of “Kikuchi-Fujimoto disease (KFD)” and “COVID-19” and then “KFD” and “heart” or “myocarditis” by searching medical journal databases written in English in PubMed and Google Scholar.

**Results:**

Only two cases of KFD after COVID infection have been described so far.

**Conclusion:**

SARS-CoV-2 infection can also be a new, potential causative agent of developing KFD.

## Introduction

The clinical course of SARS-CoV-2 infection is highly variable and data about new complications associated with this virus are presented every pandemic day. The association between COVID-19 infection and the development of autoimmune diseases has been presented by different authors. A few principal mechanisms that may contribute to the development of autoimmunity after COVID-19 infection have proposed: the ability of SARS-CoV-2 to hyper-stimulate the immune system, induction of excessive neutrophil extracellular traps formation with neutrophil-associated cytokine responses and the molecular resemblance between self-components of the host and the virus [1, 2]. Kikuchi-Fujimoto disease (KFD) was first described in 1972 in Japan, as a benign and self-limiting disease characterized by cervical lymphadenopathy and fever affecting mainly young Asian women [3]. Recent reports describe KFD as a form of histiocytic necrotizing lymphadenitis that can occur in people of all races, both sexes and in every age. In Poland, KFD is an extremally rare disease and only several cases have been described so far [4–7]. Although the etiology of KFD is undetermined, an infectious and autoimmune background has been postulated. The typical clinical presentation of KFD is unilateral posterior cervical or jugular lymphadenopathy accompanied by fever of various degrees [8]. Generalized lymphadenopathy [9], as well as lymphadenopathy limited to the mediastinum, axillary or mesenteric has also been described [3, 10, 11]. Additionally, some of the patients may complaint due to fatigue, night sweats, skin rash, arthritis, myalgia, chest, and abdominal pain (due to hepatosplenomegaly), weight loss, headache or cough [12]. The skin changes may be variable in appearance, although most commonly presents with rash, erythematous macules, papules, or plaques on the face (cheeks), upper limbs, and trunk. Leukocytoclastic vasculitis has also been reported [9]. The disease can have an acute or subacute course, evolving during a period of 2–3 weeks with spontaneous resolution of symptoms within 1–4 months in most of the cases. Heart involvement is a rare complication of KFD associated with aggressive course of the disease. Most of the patients with KFD require only supportive treatment with antipyretics and analgesics [12]. In certain cases of persisting or recurrent symptoms (3–4% of patients) glucocorticosteroids, immunoglobulins, hydroxychloroquine, cyclosporine, azathioprine or anakinra have been used [13–16].

Here we present a rare case of KFD with heart involvement after COVID-19 infection. To our best knowledge, only a few cases of COVID-19-associated KFD were published so far. Based on presented case, we summarize the clinical course of KFD and its association with autoimmune diseases, as well we discuss the potential causes of perimyocarditis in this case.

## Methods

### Case report

**Fig. 1:**
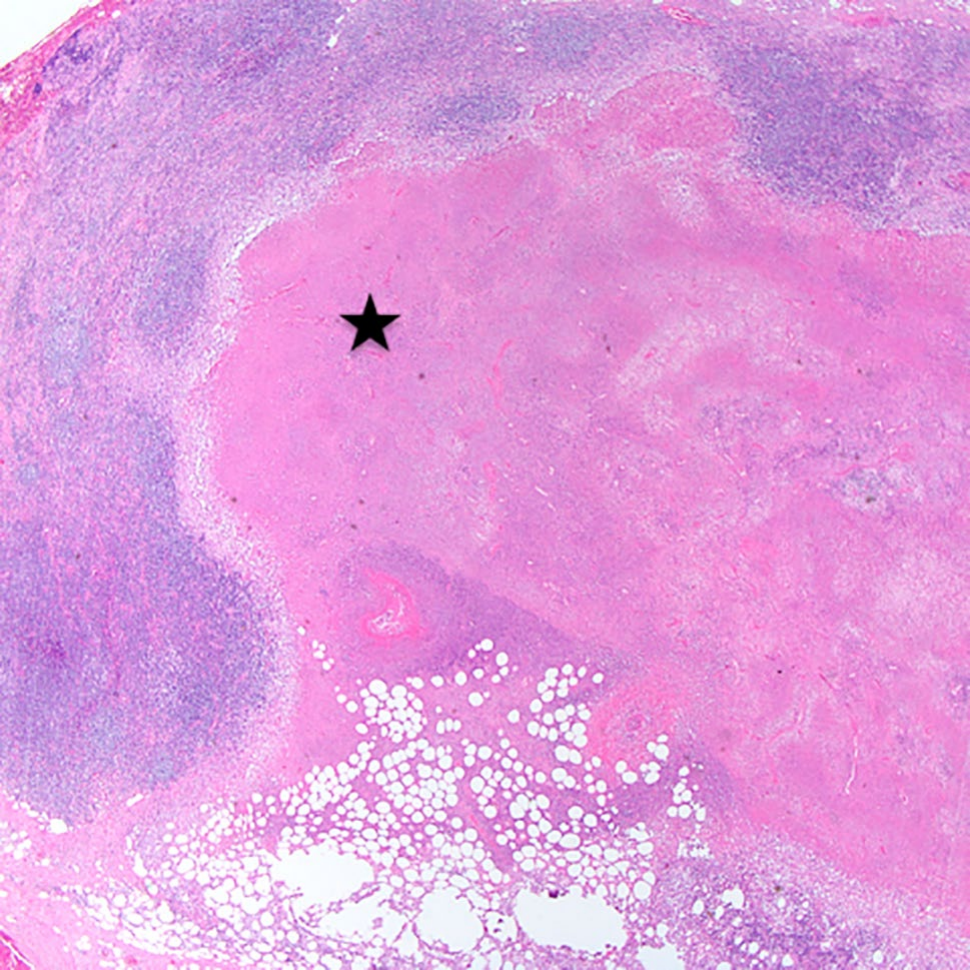
Well-circumscribed paracortical necrosis (black asterisk) in the lymph node, haematoxylin and eosin, magnification × 20

A 43-year-old Caucasian man, with no concomitant diseases, had mild form of SARS-CoV-2 infection with anosmia and ageusia for 1 day, mild fever for a couple of days. All symptoms disappeared completely within a few days. Five weeks later he was admitted to the hospital due to fever with sweats, sore throat, fatigue, dyspnea, dry cough, and skin changes for 8 days prior the hospitalization. Additionally, he reported discomfort in the right lower abdomen, discolored stools, and dark colored urine. He was treated with amoxicillin with clavulanic acid on the recommendation of his GP, and high dosages of different analgesics (paracetamol, ibuprofen, metamizol, aspirin) without medical prescription. On admission, physical examination was remarkable for dyspnea on slightest exertion, temperature 39.5 °C, regular heart rate of 97 beats/min, blood pressure of 100/75 mmHg and oxygen saturation of 94% while breathing room air. Physical examination revealed jaundice in the skin and sclerae, skin erythema on the right side of the neck, chest and back, hepatomegaly (3 cm under the rib arch), without peripheral edema. A palpable enlarged right supraclavicular lymph node—hard, about 2 cm in diameter, and several cervical lymph nodes about 1 cm were noted. His laboratory data showed leukocytosis with lymphopenia (WBC 14 G/l; lymphocytes 0.6 G/l); elevated GGTP (386 U/l), alkaline phosphatase (216 U/l) and bilirubin (7.5 mg/dl) with normal transaminase and lactate dehydrogenase levels. Inflammatory markers (CRP 281 mg/l, procalcitonin 0.9 ng/ml, ESR 62 mm/h), ferritin (3400 ng/ml) and d-dimer (4613.46 µg/L) were elevated. Troponin I level was 0.119 ng/ml (normal < 0.03 ng/ml), creatine kinase—MB level 0.6 ng/ml (< 5.2 ng/ml) and B-type natriuretic peptide (BNP) was 965 pg/ml (< 73 pg/ml). Nasopharyngeal swab for COVID-19 was negative, although immunoglobulins G (IgG) for SARS-CoV-2 were positive. Blood and urine cultures were negative. Pulmonary embolism was excluded based on the angio-CT. CT scan of the abdomen and pelvis with contrast revealed no focal changes of oncological concern or other enlarged pathological lymph nodes, but enlarged, homogenous liver without signs of cholestasis and an enlarged spleen. Echocardiography showed a globally reduced myocardial contractility and decreased ejection fraction up to 40% which in association with the clinical features indicated perimyocarditis. Due to the clinical suspicion of hemophagocytic syndrome (HLH) (fever, splenomegaly, high ferritin level), serum soluble receptor for IL-2 (11,401 U/ml, range 158–632 U/ml), NK cells (15.00%, range 6–27%), as well as bone marrow biopsy were performed. Finally, HLH was excluded (no features of hemophagocytosis in bone marrow, NK cells within normal ranges, no cytopenia, hypertriglyceridemia and hypofibrinogenemia). Based on serological tests, active infection with parvovirus B19 (past infection pattern), cytomegalovirus, hepatitis B and C, human immunodeficiency virus (HIV), Mycoplasma pneumoniae, Chlamydia pneumoniae and Yersinia enterocolitica infection were excluded. Immunological tests showed the presence of anti-nuclear antibodies (ANA-HEp2 1:2560), while the nuclear profile showed anti-DFS 70 antibodies. Autoimmune liver disease panel (AMA-M2, Sp100, PML, Gp210, LKM-1, LC-1, SLA/LP, Ro-52) was negative. Complement components C4 and C3, main immunoglobulin classes (IgG, IgA, IgM), as well as IgG4 were normal. During hospitalization, the patient was treated with amoxicillin with clavulanic acid, clarithromycin, and antipyretics. Gradually, his condition improved, body temperature normalized, and dyspnea subsided. Due to the undetermined cause of the symptoms, it was decided to obtain a lymph node for histopathological examination, which revealed necrotizing, non-granulomatous lymphadenitis suggestive for histiocytic necrotizing lymphadenitis (Kikuchi-Fujimoto lymphadenitis) (Fig. 1).

**Fig. 2:**
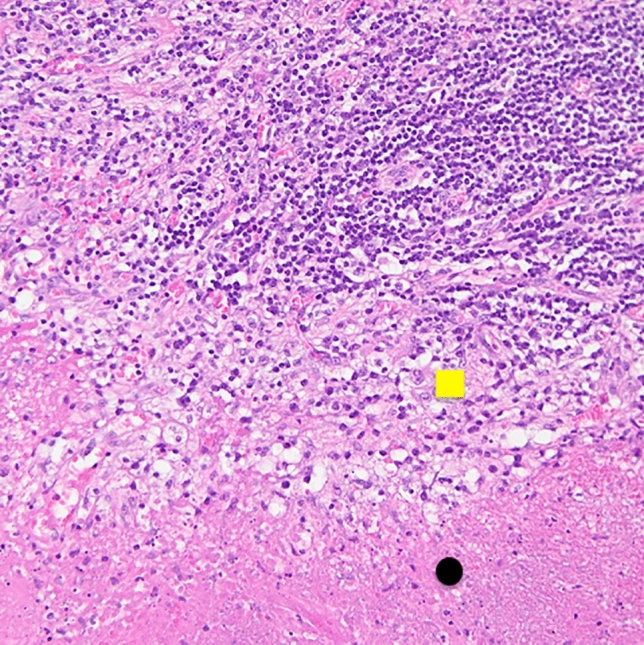
Higher magnification showing necrosis (below, black dot) with evidence of scattered nuclear dust (karyorrhectic debris) and surrounding histiocytes (in the middle, yellow square), haematoxylin and eosin, magnification × 200

Considering the course of the disease (fever, lymphadenopathy, skin lesions, hepato-splenomegaly), the results of laboratory tests and histopathological examination of the lymph node, Kikuchi-Fujimoto disease was diagnosed, most probably caused by SARS-CoV-2 infection. Towards spontaneous improvement of general condition, normalization of laboratory tests, good clinical condition of the patient, it was decided that no target treatment was necessary (the disease usually resolves spontaneously). On follow-up, 1, 4 and 7 months after discharge, he reported that all symptoms had almost completely disappeared, although physical impairment persists, and on echocardiography reduced ejection fraction (53%) with impaired global systolic heart function is still present. At present, the patient does not meet the criteria for systemic connective tissue disease, but due to the potential association of KFD disease with autoimmune diseases, it was decided that the patient should be observed in the rheumatology outpatient clinic (Fig. 2).

### Search strategy

We reviewed the literature regarding cases of “Kikuchi-Fujimoto disease (KFD)” and “COVID-19” and then “KFD” and “heart” or “myocarditis” by searching medical journal databases written in English in PubMed and Google Scholar. At first, two related case report was found [17, 18] which are summarized in Table 1. We also found one case report presenting KFD after SARS CoV-2 vaccination [19] and patient with KFD infected by SARS-CoV-2 [20]. According to second search, we identified four articles which are summarized in Table 2.

**Table 1:** Cases of Kikuchi-Fujimoto disease associated with COVID-19 infection

Author	Age, sex	Association with SARS-CoV-2	Clinical presentation	Heart involvement	ANA HEp2 positivity	treatment	outcome
Stimson [17]	17, m	2 months after infection	Cervical lymphadenopathy, parotid gland enlargement, fever, poor appetite, weight loss and fatigue	No	No	No data	Complete resolution
Racette [18]	32, m	3 months after infection	Fever, chills, neck swelling, myalgia	No	No	Prednisone	Complete resolution
Soub H [19]	18, m	10 days after receiving the first dose vaccine	Fever, cervical and axillary lymphadenopathy, nausea	No	No	Paracetamol, NSAIDS, ceftriaxone	Complete resolution
Jaseb [20]	16, f	After the KFD diagnosis	Left cervical lymphadenopathy, fever, night sweats, myalgia, weight loss, hair loss, erythematous plaques on the face, limbs, and hands	No	Yes	Prednisone	Improvement in lymphadenopathy and skin rashes

**Table 2:** Cases of Kikuchi-Fujimoto disease with heart involvement reported in the literature

Author	Year of publication	Age of presented case, sex	Clinical presentation	Association with infection or autoimmune disease	Treatment	Resolution
Silva et al. [39]	2010	24, male	Fever, arthralgia, lymphadenopathy, pericarditis, pneumoniae, acute kindey failure, hepatitis, jaundice	None	Prednisone	Improved
Joean et al. [9]	2018	18, male	Fever, night sweats, generalized lymphadenopathy, fatigue, skin vasculitis, pleural effusion, cardiomyopathy, pericarditis, hepatitis	Human herpes virus 6	Analgetics, antipyretics	Self-limiting
Chan et al. [40]	1989	38, male	Fever, lymphadenopathy, acute heart failure	None	None	Died
Quintas-Cardama et al. [41]	2003	38, female	Weight loss, fever, arthralgia, myalgia, generalized lymphadenopathy, hepatomegaly, myocardiopathy, percarditis	SLE	Antibiotic, prednison	died

## Discussion

The etiology of KFD has still to be clearly established. There are two main postulated hypotheses. Infectious background is the most suggested one, with various viral, bacterial, and parasitic pathogens suspected as causative agents for KFD [12]. There are also reports of KFD induction after breast implants [21] and with association with solid tumors [22]. SARS-CoV-2 virus may be another potential trigger of KFD. Our literature review revealed two reported cases of KFD after COVID infection, one after SARS-CoV-2 vaccination and one KFD patient with concomitant COVID-19 infection (Table 1). Our patient is third reported case with a clear time link between the SARS-CoV-2 infection and the onset of KFD.

Second hypothesis of KFD favored by some authors postulates an autoimmune background with antinuclear antibody (ANA) positivity in some patients [12]. Although, most of the KFD patient are ANA negative, KFD has been described in patients affected by different connective tissue diseases, mainly SLE (13%) [3, 12], but also Sjögren disease [6], and other connective tissue diseases [23, 24]. KFD can develop prior, simultaneously or after the onset of autoimmune disease [25]. Presented patient had high titer of ANA HEp2 antibodies which could suggest a possible autoimmune background but could also be related to a previous infection. According to the current knowledge, the presence of isolated anti-DFS70 antibodies, can serve as a diagnostic biomarker to help rule out systemic autoimmune disease [26]. Anti-DFS 70 autoantibodies were reported to be more prevalent in healthy individuals than those with autoimmune diseases such as systemic lupus erythematosus (SLE). In a long-term analysis of KFD patients, Hyun et al. found that patients who developed autoimmune diseases after KFD were more likely to have extranodal symptoms, KFD recurrence, and anti-nuclear antibody positivity [27]. There is no current literature illustrating how best to follow KFD patients regarding long-term complications and disease associations such as SLE. It seems beneficial to observe the patient for relapse or evolution of autoimmune disease. It was suggested that patients with positive serologies who experience arthralgias, skin manifestations, and weight loss are most at risk for the development of SLE [28]. Nevertheless, the patient requires further follow-up in a rheumatology outpatient clinic.

There are no KFD-specific findings in the laboratory tests. Nonspecific increased inflammatory markers, slightly elevated liver enzymes (more common in male patients with KFD [29]) or leukopenia may be present in some cases [30]. Although elevated ferritin levels may be present in KFD [31], one should always be aware of concomitance of adult-onset Still’s disease (AOSD) [32], or reactive hemophagocytic lymphohistiocytosis (HLH) [33, 34]. Data showed that patients with HLH-associated KFD may have higher serum ferritin and LDH levels compared to typical cases of KFD [33, 34]. KFD, AOSD and HLH share also other clinical similarities—fever, lymphadenopathy, rashes, or hepatosplenomegaly. The differential diagnosis of these diseases with relation to the presented case is summarized in Table 3.

**Table 3:** The differential diagnosis of adult onset Still’s disease (AOSD), hemophagocytic lymphohistiocytosis (HLH) and Kikuchi-Fujimoto disease (KFD) with relation to the presented case

	Presented case	Kikuchi-Fujimoto disease	Hemophagocytic syndrome	Still’s disease
*Patient’s symptoms*				
	Fever	Occured	Occured	Occured
	Night sweats	Occured	Irrelevant	Irrelevant
	Sore throat	Irrelevant	Irrelevant	Occured
	Fatigue	Occured	Occured	Irrelevant
	Dyspnea	Irrelevant	Irrelevant	Irrelevant
	Dry cough	Irrelevant	Irrelevant	Irrelevant
	Skin changes	Occured	Occured	Occured
	Abdomen pain	Occured	Occured	Irrelevant
	Discolored stools	Irrelevant	Irrelevant	Irrelevant
	Dark colored urine	Irrelevant	Irrelevant	Irrelevant
*Physical examination*				
	Jaundince	Irrelevant	Occured	Irrelevant
	Skin erythrema	Occured	Occured	Occured
	Hepatomeghaly	Occured	Occured	Occured
	Enlarged (supraclavicular) nodes	Occured	Occured	Occured
*Patient’s laboratory data*				
WBC	14 G/l	Elevated	Decreased	Elevated
Lymphocytes	0.6 G/l	Irrelevant	Pancytopenia	Irrelevant
LDH	170 U/l	Elevated	Elevated	Elevated
GGTP	386 U/l	Irrelevant	Elevated	Elevated
ALP	216 U/l	Irrelevant	Elevated	Elevated
Bilirubin	7.5 mg/dl	Irrelevant	Elevated	Elevated
ESR	62 mm/h	Elevated	Irrelevant	Elevated
CRP	281 mg/dl	May be elevated	May be elevated	Elevated
Procalcytonin	0.9 ng/ml	Irrelevant	May be elevated	Irrelevant
Ferritin	3400 ng/ml	Irrelevant	Elevated > 500 ug/l	Elevated
D-dimer	4613,46 ug/L	Irrelevant	May be elevated	Irrelevant
Serum soluble receptor for IL-2	11,401 U/ml	Irrelevant	Elevated	Irrelevant
NK cells	0.14 G/l	Irrelevant	Decreased	Irrelevant
Blood culture	Negative	Irrelevant	May be positive	Irrelevant
Urine culture	Negative	Irrelevant	May be positive	Irrelevant
Triglicerydes	232 mg/dl	Irrelevant	Hypertriglycerydemia	Irrelevant
Fibrinogen	7.44 G/l	Irrelevant	Hypofibrinogemia	Irrelevant
HIV-1/2 Ag/AB	Negative	Irrelevant	Irrelevant	Irrelevant
CMV IgG, IgM	IgG (–), IgM (−)	Irrelevant	Irrelevant	Negative
Parvovirus B-19 IgG, IgM	IgG (+)–140 IgM (–)–< 0.1	Irrelevant	Irrelevant	Irrelevant
ANA-Hep2	1:2560	Generally negative	Irrelevant	Negative
Nuclear profile	Anti-DFS-70 antibodies	Irrelevant	Irrelevant	Negative
Complement component—C3, C4	C3—2.36 G/l C4—0.2 G/l	Irrelevant	Irrelevant	Negative
Immunoglobulin classes – IgG4	0.54 G/l	Irrelevant	Irrelevant	Negative
*Patient’s imaging tests and biopsy*				
	Angio-CT—pulmonary embolism excluded	Irrelevant	Irrelevant	Irrelevant
	CT scan of the abdomen and pelvis—enlarged, homogenous liver without sign of cholestasis and enlarged spleen; no focal changes of oncological concern; no enlarged pathological lymph nodes	Occured-hepatosplenomegaly	Occured-hepatosplenomegaly—ascites, gallbladder wall thickening, increased periportal echogenicity, lymphadenopathy, and pleural effusion	Irrelevant
	Echocardiography—globally reduced myocardial contractility and decreased EF up to 40%; clinical features indicated perimyocarditis	Occured-rarely involved extranodal sites include myocardium	Irrelevant	Irrelevant
	Biopsy of bone marrow—no features of hemofagocytosis	Irrelevant	Hemophagocytosis—Must have tissue demonstration from lymph node, spleen, or bone marrow without evidence of malignancy	Irrelevant
	Biopsy of cervical lymph nodes—necrotizing, non-granulomatous lymphadenitis	Occurred-necrotizing phase—extensive necrosis that may destroy the normal architecture of the lymph node, histocytes—crescent-shaped nuclei, karyorrhexis—histiocytes and macrophages containing phagocytized debris from degenerated lymphocyte, absent neutrophils and granulomas	Hemophagocytosis	Irrelevant

The diagnosis of KFD is based on excisional lymph node biopsy. Histopathological evaluation is essential not only for proper diagnosis but also for the exclusion of other clinically similar entities: lymphoma, metastasis, tuberculous adenitis. Typical histologic features of KFD include the presence of areas of necrosis with a high degree of karyorrhexis (necrotizing lymphadenitis), but absence of neutrophils and eosinophils. Histiocytes, immunoblasts, and plasmacytoid dendritic cells can be identified in the surrounding periphery [35]. Type of infiltrating cells (CD8+ T cells prevalence), absence of hematoxylin bodies, or myeloperoxidase co-expression by CD68 histiocytes in lymph node biopsies from patients with Kikuchi-Fujimoto can be helpful in differentiation between SLE and malignant lymphoma [36]. Neutrophils are typically absent, which allows to differentiate KFD from bacterial lymphadenitis.

Although most KFD patients have a benign clinical presentation, in some cases the disease may have more aggressive course with severe complications like HLH [37], pulmonary hemorrhage, acute heart failure or hemolytic anaemia [12, 38]. Cases of KFD with heart involvement have been rarely reported. We were able to find only four reports in English language (Table 2). Silva et al. presented a 24-year-old man with severe clinical manifestation of KFD such as pneumoniae, hepatosplenomegaly, acute renal failure and pericarditis with cardiac tamponade [39]. Joean et al. showed an 18-year-old man with high fever and reduced ejection fraction of a left heart with pericardial effusion [9]. Chan [40] and Quintas-Cardama [41] presented two fatal cases of KFD who had heart involvement. A review of the literature has highlighted how rare cardiac involvement is in KFD. This forced us to consider other potential causes of heart involvement in our patient. We focused on cardiac involvement in the course of SARS-CoV-2 infection which was another potential cause of myocarditis in the presented case. Most reports concerning cardiac manifestations of COVID-19 describe patients with active infection [42–44]. However, there are some case reports presenting patients with symptoms suggestive for myocarditis occurring only after resolution of the SARS-CoV-2 infection [45]. The newest data suggest that myocardial injury is common in COVID-19 patients and occurs irrespective of the severity of the initial presentation. In meta-summary of 51 cases of myocarditis and SARS-CoV-2 infection, there was 1 patient recently recovered from COVID-19 pneumonia 3 weeks prior to presentation with myocarditis primary presented by Sardari et al. [46, 47]. In the study of Kamal et al. focused on post-COVID-19 manifestations, 1.4% of patient had symptoms of myocarditis [48]. It is not possible to determine what was the direct cause of myocarditis in the presented patient. Both potential causes of KFD and SARS-Cov-2 are probable.

We are fully aware that in the case presented, the association of KFD with SARS-CoV-2 infection cannot be unequivocally demonstrated. As the patient had no symptoms of connective tissue disease, we think it is reasonable to assume that a previous COVID-19 infection was the triggering factor of the KFD. Also, the aetiology of the cardiac lesions cannot be clearly established as mentioned above.

## CONCLUSION

KFD is a great mimicker and pose a diagnostic dilemma. The differential diagnosis is based on the main causes of lymphadenopathy and fever and include mainly lymphoma, various types of infectious agents, and autoimmune diseases. To aid medical practitioners to identify this rare disorder, a diagnostic flow chat was proposed by Xu et al. [49]. SARS-CoV-2 infection can also be a new, potential causative agent of developing KFD.
